# Experimental study of the quantification of indocyanine green fluorescence in ischemic and non-ischemic anastomoses, using the SERGREEN software program

**DOI:** 10.1038/s41598-022-17395-6

**Published:** 2022-07-30

**Authors:** X. Serra-Aracil, A. García-Nalda, B. Serra-Gómez, A. Serra-Gómez, L. Mora-López, A. Pallisera-Lloveras, V. Lucas-Guerrero, S. Navarro-Soto

**Affiliations:** 1 Coloproctology Unit, General and Digestive Surgery Service, Parc Taulí University Hospital, Departament de Cirurgia, Universitat Autònoma de Barcelona, Parc Taulí s/n, 08208 Sabadell, Barcelona Spain; 2 Taulí Research and Innovation Institute I3PT, Parc Taulí University Hospital, Departament de Cirurgia, Universitat Autònoma de Barcelona, Parc Taulí s/n, 08208 Sabadell, Barcelona Spain

**Keywords:** Colon cancer, Rectal cancer, Fluorescence imaging

## Abstract

Tissue ischemia is a key risk factor in anastomotic leak (AL). Indocyanine green (ICG) is widely used in colorectal surgery to define the segments with the best vascularization. In an experimental model, we present a new system for quantifying ICG fluorescence intensity, the SERGREEN software. Controlled experimental study with eight pigs. In the initial control stage, ICG fluorescence intensity was analyzed at the level of two anastomoses, in the right and in the left colon. Control images of the two segments were taken after ICG administration. The images were processed with the SERGREEN program. Then, in the experimental ischemia stage, the inferior mesenteric artery was sectioned at the level of the anastomosis of the left colon. Fifteen minutes after the section, sequential images of the two anastomoses were taken every 30 min for the following 2 h. At the control stage, the mean scores were 134.2 (95% CI 116.3–152.2) for the right colon and 147 (95% CI 134.7–159.3) for the left colon (p = 0.174) (Scale RGB—Red, Green, Blue). The right colon remained stable throughout the experiment. In the left colon, intensity fell by 47.9 points with respect to the pre-ischemia value (p < 0.01). After the first post-ischemia determination, the values of the ischemic left colon remained stable throughout the experiment. The relative decrease in ICG fluorescence intensity of the ischemic left colon was 32.6%. The SERGREEN program quantifies ICG fluorescence intensity in normal and ischemic situations and detects differences between them. A reduction in ICG fluorescence intensity of 32.6% or more was correlated with complete tissue ischemia.

## Introduction

In recent years, technological advances have allowed the development of many new instruments for performing more effective and safer surgeries.

In laparoscopic surgery, indocyanine green (ICG) is widely used to improve the evaluation of tissue viability. ICG is a solid compound that emits infrared light with a wavelength of 830 nm when it is stimulated by an infrared light source at 806 nm^1^. However, its applicability has been limited by the subjectivity inherent in its evaluation; despite attempts of standardization, its assessment remains highly observer-dependent^2^. In colorectal surgery ICG is mainly used to study the vascularization of the intestinal segments for creating anastomoses. As is well known, poor intestinal vascularization can lead to ischemia, which may cause perforation due to necrosis and also anastomotic leak^3–5^.

Anastomotic leak is one of the most feared postoperative complications of abdominal surgery with intestinal anastomosis, due to the high associated morbidity and mortality. Its incidence varies considerably, being between 1 and 30% when it comes to colonic anastomoses^6,7^.

In order to perform better anastomosis and reduce anastomotic leak, surgeons support their decisions on different items and parameters where ICG is gaining a major role^8^.

The aim of the present experimental study was to evaluate the clinical applicability of the SERGREEN program, a genuine software program developed by our research team which provides an objective method for quantifying ICG and reflects the state of tissue vascularization.

The main objective was to assess the differences in tissue perfusion around the anastomosis in two intestinal segments: one vascularized and one ischemic through the application of this software. If it is capable of traduce those changes, it can help us to identify areas of interest during future operations in human beings.

Colonic site of section, mesocolon dissection and colonic anastomosis must be well perfused if we want that they work properly. Application of this kind of softwares can help us to do it in a better way and optimize our surgical procedures and surgical decisions.

## Material and methods

### Ethical statement

The study was approved by the Ethics Commission for Animal and Human Experimentation of the Autonomous University of Barcelona, to which our hospital is affiliated (Ref. CEEAH 10; 26-2016). All methods were carried out in accordance with relevant guidelines and regulations.

### Study design

Experimental, controlled, prospective study in animals. After laparoscopic surgery and ICG administration, the vascularization of the colon was quantitatively determined using instantaneous images with the SERGREEN program. This study followed the ARRIVE (Animal Research: Reporting of In Vivo Experiments) guidelines.

This study is the pre-IDEAL framework phase in the SERGREEN software project^9,10^

Eight farm pigs (Large-White breed: four males and four females, weight 30–35 kg and age 2–4 months) were used. They were imported from SPECIFIC PIGS S.L. with all the required health and transport documentation.

The left colon and the right colon of each pig will be analyzed and observed always the same point, at the same time and at the same intraabdominal pressure after ICG administration.

### Experimental procedures

Animals were operated upon at the Experimental Surgery Unit of the Parc Taulí University Hospital by the hospital’s colorectal surgery team, with support from the veterinary team. The surgery was performed under general anesthesia in accordance with the center’s animal anesthesia protocol.

Sedation was performed by intramuscular injection of zolethyl 100 (2 mg/kg tiletamine + 2 mg/kg zolazepam), xylazine (2 mg/kg) and atropine (0.01 mg/kg). The atrial vein was channeled and propofol injected in bolus (1.5 mg/kg). Once the animals were sedated and relaxed, orotracheal intubation was performed. Sedation and analgesia were maintained by continuous infusion pump using propofol and fentanyl, and fluid therapy was maintained with Ringer’s lactate solution.

Animals’ vital signs were recorded at baseline, after anesthesia, analgesia and relaxation, and prior to surgery. They were maintained stable throughout the procedure, with heart rate ranging between 70 and 95 beats per minute and mean blood pressure between 60 and 80.

The laparoscopic equipment used was the IMAGE 1 H3-Z FI and IMAGE 1 HUB HD Camera Control Unit SCB (Karl-Storz®). Pictures were taken in STANDARD, SPECTRA A and ICG mode. Images were recorded from the time just prior to the infusion until the end of the procedure. The analysis of all the pictures were done in ICG mode under SPECTRA A layer.

Two groups were created for the study of the anastomoses. The control group underwent two phases of surgery, one open and one laparoscopic; in the second group, which also underwent open and laparoscopic surgery, an ischemic segment was created at the level of the left colon, while the anastomosis of the right colon was left undisturbed.

All the experimental procedures were carried out by the same members of the research team, at the same ICG dose, with the same observation time and the same light source and laparoscopic optic exposure. In view of the drug’s technical specifications and elimination half-life, a 30-min interval was observed between each determination and each new administration to ensure adequate clearance of the blood flow.

### Control group

#### Open phase

A midline laparotomy was performed and the right colon and left colon segments of interest were located. The second loop of the right colon (spiral) and the segment of the left colon located in the center of the arcade of the inferior mesenteric artery and vein were selected. Both colon segments were sectioned and anastomosed again using a running end-to-end 3/0 silk suture.

After creation of the anastomoses, the laparotomy was closed with a running, hand-sewn 0 silk suture.

#### Laparoscopy phase

10-mm laparoscopic trocars were inserted at the level of the mesogastrium, to the right of the midline laparotomy incision, to allow introduction of the laparoscopic camera. A 10-mm trocar on the right quadrant and a 5-mm trocar on the left quadrant were used to introduce the laparoscopic equipment. Intra-abdominal pressure was kept constant with a 12 mmHg self-regulating insufflator.

**Figure 1:**
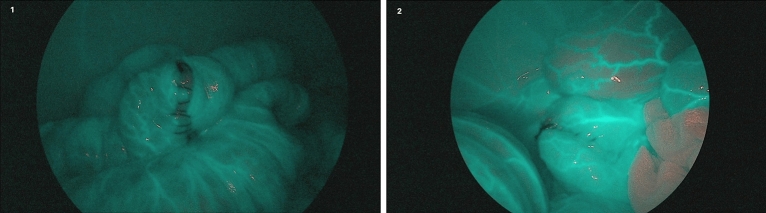
Laparoscopic image in Spectra A + ICG mode of running suture manual end-to-end anastomosis of the right colon (1), without arterial manipulation, and of the left colon (2), without arterial manipulation.

The ICG was injected at a dose of 0.2 mg/Kg through the atrial vein and the recording of video and images began. Starting two minutes after contrast infusion, standard and Spectra A images were taken of both the right and left colon anastomoses (Fig. 1).

### Post-ischemia group

#### Open phase

Thirty minutes after the administration of the first dose of ICG, the abdominal cavity was accessed again through the mid-line laparotomy performed earlier. At the level of the anastomosis of the left colon, the arcade of the mesenteric artery and vein was divided less than 5 cm proximal and 5 cm distal from the anastomosis, creating an area of about 10 cm without vascularization.

After dividing the artery, the midline laparotomy was closed again using a running suture 0-silk. The right colon anastomosis is not touched, keeping it undisturbed all along the experiment. We waited 15 min to ensure complete ischemia of the left colon segment.

#### Laparoscopy phase

A 0.2 mg/Kg dose of ICG was again administered through the atrial vein. Starting two minutes after contrast infusion, Standard and Spectra A images were taken of the two anastomoses (right colon and ischemic left colon).

**Figure 2:**
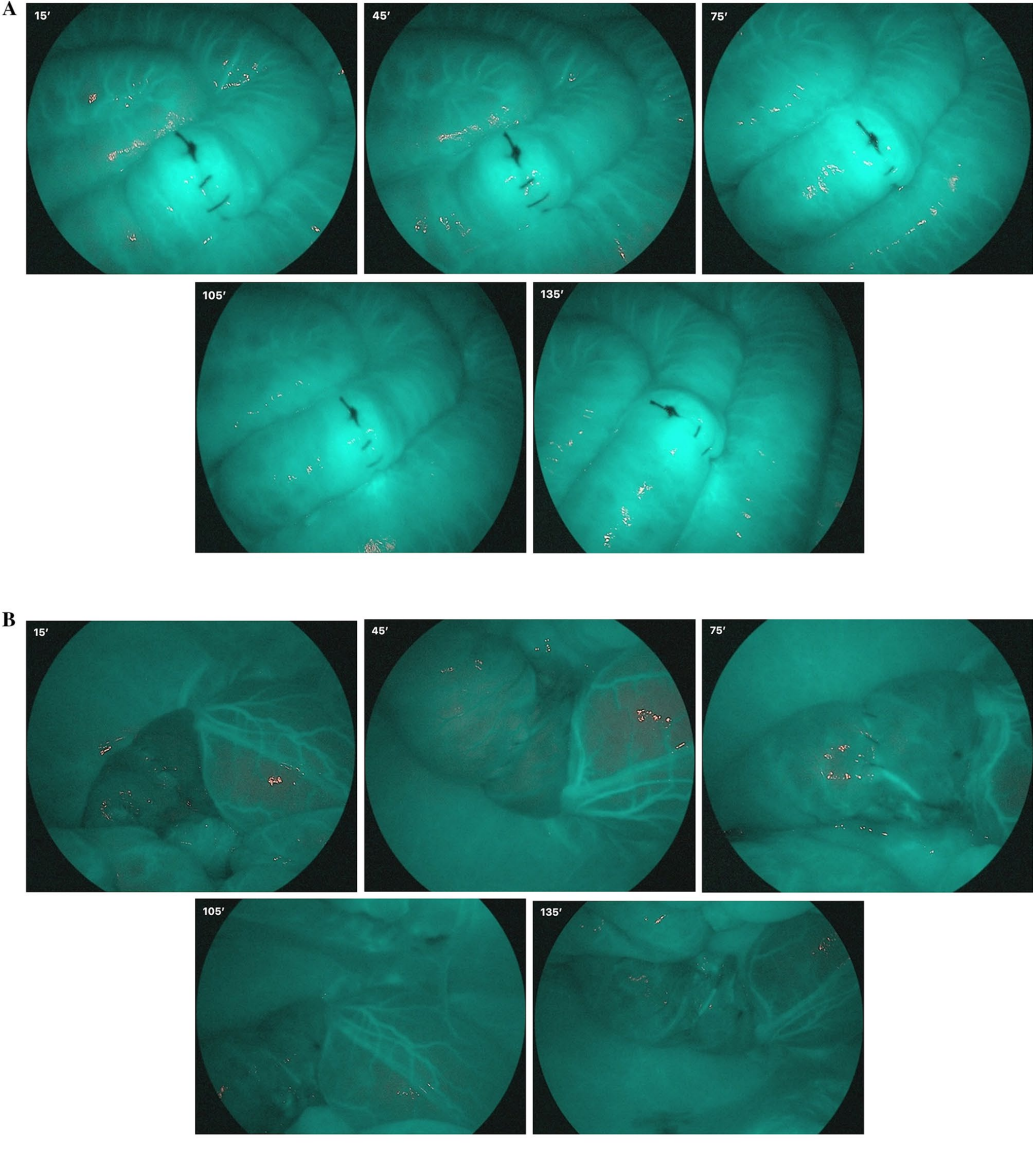
(**A**) Evolution in images of the right colon after sectioning of the inferior mesenteric artery. Images are taken at 15, 45, 75, 105 and 135 min after the arterial section. Vascularization remains uniform over time. (**B**) Evolution in images of the left colon after sectioning of the inferior mesenteric artery. Images are taken at 15, 45, 75, 105 and 135 min after the arterial section. Ischemia remains uniform over time.

This procedure was repeated every 30 min, under the same conditions, up to a maximum of 120 min (135 min after the vascular section) (Fig. 2A,B).

### Experimental outcomes: the SERGREEN software

The results were analyzed using the SERGREEN program, measuring the segments of interest in areas of uniform vascularization two minutes after ICG injection. The program was created using the MATLAB-2014b software tool (R2014b, 8.4.0. 150421. 64-bit -maci64- September 15, 2014).

The images obtained during the experimental procedure are recorded in *.jpg format with the laparoscopy equipment. This format is processed by the computer program and broken down into three matrices according to the RGB (Red, Green, Blue) color model. Each cell in each matrix corresponds to a pixel with its respective intensity in these three colors. All pixels obtain 24 bits (eight in each matrix) with the information from the three colors.

On the decimal scale, eight bits are represented from 0 to 255, and this is how these values are represented. By overlapping the three channels from the RGB image, we obtain the color seen in the original image. SERGREEN processes the matrix corresponding to green and compares it with the other two in order to determine the predominant color.

Pixels whose predominant color is not green are discarded. In this way, false readings caused by reflections are avoided, since white is the composition of the three colors at their maximum value (R: 255, G: 255 and B: 255).

The program’s main tool allows users to mark a rectangle wherever they like in the image and obtain statistical information on the intensity of the green in that region. All the pixels corresponding to the green channel within the specified rectangle can be exported into an Excel file. Another document is generated to check the image selected, as well as a boxplot chart, a histogram, and a summary of the central tendency values.

### Experimental outcomes: image processing and analysis of the results obtained with SERGREEN

This measurement was carried out in 10 different areas with 10 × 10 pixel squares. A sampling of different areas of interest was generated within the same intestinal segment to ensure its correct representation. A matrix was obtained that included the eight pigs, all their intestinal segments and their 10 measurements according to area of interest and for each time of observation, including all the pixels contained in the squares.

**Figure 3:**
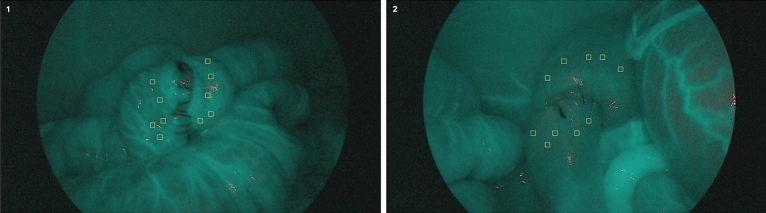
Ten areas of study of vascularization in the right colon (1) and in the left colon (2) using 10 × 10 pixel squares, highlighted in yellow circles. Predefined squares are applied over areas of uniform vascularization.

At the level of the anastomosis, between 1–2 cm proximal and distal to the suture line, 10 areas of interest were recorded in all the images obtained of both the right and left colon, avoiding the areas that correspond to visible vessels (Fig. 3).

### Statistical and calculation methods

The ICG fluorescence intensity obtained applying the SERGREEN program to the images of interest was taken as the main variable. The Kolmogorov–Smirnov test was applied to check the normal distributions.

Results are expressed as means for each segment (the sample mean of the pigs for each segment and observation time).

The student “t” test was used for comparisons between two means, while the ANOVA test was used to compare different means, since the distributions were comparable to normal. The decreases in the intensity of the ICG after generating the ischemic area are expressed in absolute and relative terms.

The absolute mean ICG saturation decrease (AMISD) was calculated as the mean ICG saturation of the pre-ischemic left colon (MISPreI = Mean ICG Saturation PreIschemia) minus the mean ICG saturation of all post-ischemic left colon saturation determinations (MISPostI = Mean ICG Saturation PostIschemia).

$$AMISD= MISPreI-MISPostI$$

We describe the relative mean ICG saturation decrease (RMISD) which is calculated as the unit minus the division of the non-vascularized area (MISPostI) with respect to the vascularized area (MISPreI) of the left colon. This can be expressed as a percentage of relative decrease by multiplication by 100.

$$RMISD=\left(1-\frac{MISPostI}{MISPreI}\right)\times 100$$

## Results

Data from the eight pigs were recorded. Data from the inferior mesenteric artery section in pig 3 and from minute 105 post-ischemia in pigs 2 and 8 were excluded due to cardiorespiratory arrest in these three cases.

The results for the two groups are presented below and the pre-ischemia and post-ischemia results are compared.

### Control group

#### Right colon

The mean intensity of the right colon was 134.2 (95% CI 116.3–152.0). The mean pre-ischemia ICG values in the right colon for each pig are displayed in Table 1.

**Table 1:** Summary of the mean saturations in the right colon and left colon during the control period, prior to the section of the inferior mesenteric artery and vein.

ICG saturation		
Nº pig	Right colon pre-ischemia	Left colon pre-ischemia
1	152.57	156.90
2	159.43	137.10
3	143.42	167.29
4		
5	103.08	137.39
6	121.19	130.66
7	130.77	143.49
8	128.72	155.99

#### Left colon

The median intensity of the left colon was 147 (95% CI 134.7–159.3). The mean pre-ischemia ICG values in the left colon for each pig are also displayed in Table 1.

There were no statistically significant differences between the mean ICG fluorescence intensity of the right colon and the left colon prior to the creation of ischemia; the two segments were comparable (*p* = 0.174).

### Post-ischemia group

#### Right colon

**Figure 4:**
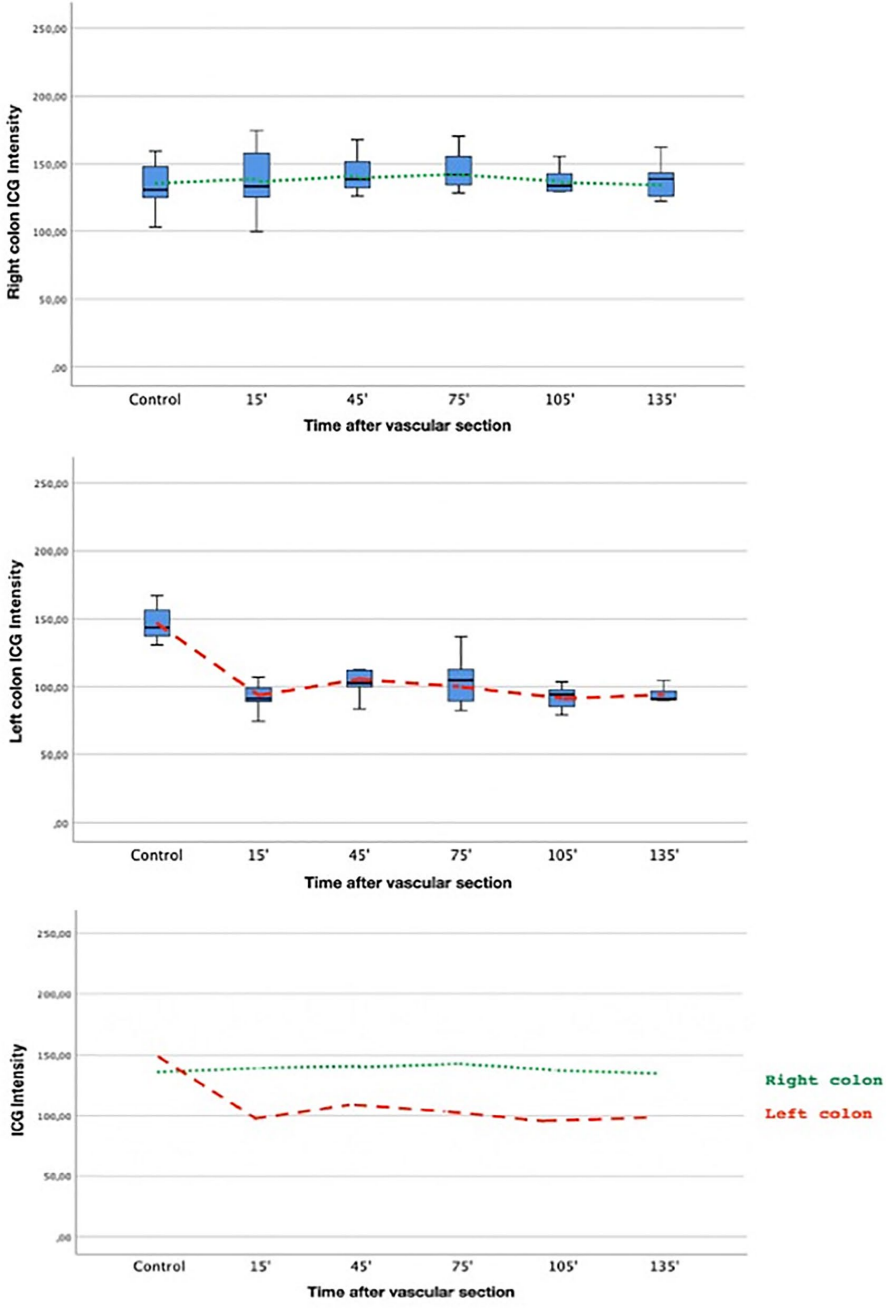
Boxplot showing the evolution over time from baseline prior to the section of the IMA to minute 135 after its section and that of the vein at the level of the right (1) and left (2) colon. The overlap of the two evolutions over time can be seen in graph 3, where the solid line represents right colon and the dashed line the left.

In the right colon, no differences were detected among the different observation times during the procedure; the scores remained stable throughout all the experiment (mean: 140; 95% CI 134.2–145.7. ANOVA p = 0.889) (Fig. 4).

The mean values for the right colon for each pig and each observation time are displayed in Table 2.

**Table 2:** Summary of the means for each pig and observation time in the left colon during the period of intestinal ischemia.

Right colon ICG saturation						Left colon ICG saturation				
Nº of pig/time after cutting the IMA (minutes)	15	45	75	105	135	15	45	75	105	135
1	163.80	156.84	170.54	155.68	162.36	74.27	102.50	112.06	78.92	89.75
2	174.84	167.98	165.34			126.93	135.27	136.91		
3										
4	126.81	134.46	136.31	129.70	126.07	90.72	83.40	82.15	85.21	90.04
5	99.80	126.00	128.37	133.71	122.30	87.42	97.17	84.61	97.46	90.70
6	123.98	129.96	132.34	129.63	138.74	107.12	102.60	104.77	103.61	104.70
7	133.30	138.58	141.98	142.63	143.20	91.04	111.16	93.94	94.16	96.59
8	151.93	146.11	145.57			91.04	112.39	112.99		

The student “t” test comparing the control observation of the right colon and each of the subsequent observation times did not show statistically significant differences (Table 3).

**Table 3:** Summary of the means of each observation time on the right colon and comparison of each one with the control time: all comparisons presented statistically significant differences.

Normal right colon	Vascular condition	N	Mean	Estándar deviation	Sig. (bilateral)	95% CI of the difference	
	Pre-ischemia	7	134.1686	19.31546		Lower	Upper
Right colon 15' after cutting IMA	Post-ischemia	7	139.2086	25.85135	0.687	− 31.61512	21.53512
Right colon 45' after cutting IMA	Post-ischemia	7	142.8471	15.14686	0.368	− 28.89268	11.53554
Right colon 75' after cutting IMA	Post-ischemia	7	145.7786	16.24581	0.247	− 32.39476	9.17476
Right colon 105' after cutting IMA	Post-ischemia	5	138.2700	11.07933	0.681	− 25.65616	17.45330
Right colon 135' after cutting IMA	Post-ischemia	5	138.5340	15.87755	0.688	− 27.87441	19.14355
Normal left colon	Pre-ischemia	7	146.9743	13.29278		Lower	Upper
Left colon 15' after cutting IMA	Post-ischemia	7	95.5057	16.83993	0.000	33.80077	69.13637
Left colon 45' after cutting IMA	Post-ischemia	7	106.3557	15.99919	0.000	23.48885	57.74829
Left colon 75' after cutting IMA	Post-ischemia	7	103.9186	19.07877	0.000	23.90663	62.20480
Left colon 105' after cutting IMA	Post-ischemia	5	91.8720	9.82825	0.000	39.41066	70.79391
Left colon 135' after cutting IMA	Post-ischemia	5	94.3560	6.42648	0.000	38.17602	67.06055

#### Left colon

In the left colon, differences were detected between the mean control observation and the mean of all post-ischemic determinations (*p* < 0.01) (Fig. 4).

No differences were detected between observation times within the ischemia period, which remained stable throughout the experiment after dividing of the vascular arcade (mean: 99.1; 95% CI 93.5–104.7; ANOVA *p* = 0.385). The mean values for the left colon in each pig and each observation time are displayed in Table 2.

Comparison of the control observation in the left colon with each of the subsequent observations throughout the experiment revealed statistically significant differences (Table 3).

The absolute mean decrease in the ICG saturation of the left colon was 47.9 points (95% CI 35.2–60.6) and the relative decrease between ischemia and non-ischemia was 32.6%.

$${{A}}{{M}}{{I}}{{S}}{{D}}= MISPreI-MISPostI= 146.9743- 99.0839=47.8904 \simeq 47.9$$

$${{R}}{{M}}{{I}}{{S}}{{D}}=\left(1-\frac{MISPostI}{MISPreI}\right)\times 100=\left(1- \frac{99.0839}{146.9743}\right)\times 100= 32.5842001\simeq 32.6\boldsymbol{ }\boldsymbol{\%}$$

## Discussion

Over the last decade, the use of ICG to detect alterations in intestinal perfusion and to reduce its inherent complications has increased. In the reports published in the literature, the aim has always been to try to detect these areas of hypoperfusion. However, the evaluation of the images has always been dependent on the subjective opinions of the raters.

In the search for greater objectivity, ordinal scales have been described for the assessment of fluorescence intensity. Sherwinter et al.^11^ graded the intensity based on the absence of green and/or the degree of apparent perfusion.

To avoid this subjectivity in the interpretation of ICG images, various computer software programs have been designed to try to quantify its intensity. The VR-RENDER PERFUSION® used by Diana’s team^12^ uses a light rating system based on an arbitrary scale assigned by the developer with an absolute maximum and a minimum equivalent to 0. The Spy-Q software (LifeCell Corp.®), used by Moyer et al.^13^, marks areas of maximum perfusion and compares them with other areas of lower intensity, generating a percentage scale from higher to lower degree of signal and correlating it with vascularization.

Another software available in the market for ICG fluorescence intensity quantification is Quest™. Quest medical imaging, from OLYMPUS® is a full medical device that includes the hardware (laparoscopic tower and light source with double channel for visible and infrared light) and the software (Spectrum 2.0®)^14^ which is designed to give a value to the amount of infrared light that arrives to the camera. The principle behind this system is to traduce fluorescence intensity to a number of lumens that the camera detects from the tissue of interest. We call lumens (lm) to a measure that express the intensity of visible light that is perceived by the human eye, which calculated from the candela (cd), the international system unit for light intensity quantification ^15^. The software has the property to give information in real time and it is useful in laparoscopic surgery and in open procedures aswell. The difference with our software is that this one has not an upper limit, there is not a maximum of lumens that it can quantify, so it gives an open scale.

Other softwares use closed scales, as IC-CALC, from Pulsion Medical Systems. This one does not focus on the typical green color but on a grey scale^16^. It analyses the intensity of infrared images using a scale that ranges from withe (high grade of contrast) to black (absence of contrast). The problem of it is that it can include certain degree of infrared light that does not correspond completely to the ICG related fluorescence. This is because the camera detects infrared light and it can be the result of the endogenous infrared emission plus the exogenous infrared fluorescence related to the ICG, which is the predominant in this kind of procedures.

What we pretended creating our own software was to implement the advantages of the previous in one program that can give exactly the information that we want, a closed rang system based on an international scale (RGB) that traduces what the surgeon sees when he looks the screen.

Focusing on the results, prior to the section of the inferior mesenteric artery and vein, the two anastomoses were analyzed to establish that the data allowed their assessment as comparable segments. No differences were detected between them. After induction of ischemia, we carried out the analysis separately in the right and left colon.

Two main points should be highlighted with regard to the data obtained for the right colon. First, they were obtained in a uniform manner and without any statistically appreciable variability. Fluorescence intensity levels remained stable throughout the observation time, thus demonstrating their usefulness as control values. Second, the time interval of 30 min between measurements and after each administration of ICG allowed the organism to eliminate the compound from the bloodstream, without any signs of accumulation in the various observations.

Analysis of the images of the left colon after ischemia induction shows that SERGREEN is able to reflect the significant reduction in ICG fluorescence intensity in the segment of interest. Once the ischemia was established (from 15 min after induction), there were no differences among the measurements over the two hours of observation. This means that once ischemia has been established, and if there are no changes that reverse it, it remains stable. From this point onwards, the measurement can be made at any time.

In the left colon the mean pre-ischemic intensity value fell from 146 points (95% CI 134.7–159.3) to a mean value of 99.1 (95% CI 93.6–104.4) counting all the measurements performed during the period of ischemia. That is, the absolute mean difference after ischemia induction was 47.9 points (95% CI 35.2–60.6).

We also obtained a relative percentage difference of 32.6%, as described above. This relative value gives an idea about tissue devascularization and its relation with tissular necrosis. It cannot be taken as a value of reference or a dogma, but it is an approximation of what we are looking for in the future, an item that help us to take decisions based on a patient-depending parameter.

Analyses of retrospective series of patients using subjective analog scales have shown an association between the use of ICG and anastomotic leak. Otero-Piñeiro et al.^17^ reported that the use of ICG modified the colon resection point in more than a quarter of the patients, and thus helping to reduce the rate of anastomotic leak. Impellizzeri et al.’s^18^ findings support this hypothesis, suggesting that this association could be demonstrated through randomized studies.

If changes of this kind are already noted with the current use of ICG in routine clinical practice, our hypothesis is that an objective system such SERGREEN can help to optimize anastomoses and reduce the risk of anastomotic leak.

Even so, a series of steps are still required before our software can be applied clinically. First, we need to establish the normal values of the various segments of the colon and small intestine (especially the terminal ileum).

To be able to do this, we must eliminate systematic biases. We need to know the optimal time to read the images after injection of ICG at the recommended dose of mg/Kg of the patient, in the same conditions of intra-abdominal pressure and luminosity^19^. Different doses of ICG implies different amount of compound circulating, so it is important to use always the same dosage to avoid that bias.

Subsequently, the optimal reading distance of the images must be determined. Under these conditions, after repeated observation, we will be able to obtain the baseline intensity values of the various intestinal segments. These values will allow us to obtain cut-off points for making intraoperative clinical decisions based on their sensitivity and specificity.

The intestinal vasculature of the pig is larger in relation to the intestinal wall than in humans, and the vascularized areas are more visible. These areas were not included so as to avoid variability in the fluorescence intensity obtained and also to avoid the high limits in the ICG intensity values close to 255 which distort the image.

The value of the relative decrease in ischemia with respect to non-ischemia, 32.6%, is an initial approximation that can help us in decision-making. Extrapolating this value obtained in an experimental model in pigs to routine clinical practice could help us to decide whether an intestinal segment is at risk of ischemia.

The main aim of the application of SERGREEN in colorectal surgery is to improve the viability of anastomoses. It is well known that correct healing is a multifactorial issue. However, adequate vascularization is one of the most important factors. SERGREEN is particularly useful in anastomoses in which the risk of leak is highest, that is, low colorectal anastomoses that require a protective ileostomy. The combination of technical expertise and the optimal vascularization values provided by SERGREEN could well benefit the patient by avoiding the need for an ileostomy, with the complications and significant reduction in quality of life that this procedure may entail^20,21^.

The SERGREEN program can be applied to many other fields of abdominal surgery. In emergency surgery, it can guide the decision whether to resect an ischemic segment after a strangulated hernia or intestinal occlusion. Other possible applications include the study of the sentinel lymph node in cancer surgery^22^, the viability of free grafts in plastic surgery^13^ and the measurement of ICG in liver metastases compared with other space-occupying lesions^23^.

A limitation of the study is the fact that the distance from the camera to the tissue was not controlled. Other limitations can be the different camera degrees during laparoscopic surgeries. In our study a single 0-degree camera was used in all the procedures so this does not affect the final results or its interpretation. Even so, all procedures were carried out by the same team of surgeons and all images were taken at the same observation time. This circumstance and the animal experimental setting suggest that the results are reliable, given the uniform nature of the readings and the fact that any errors present will be systematic.

Another limitation of our method is that it is based only on fluorescence intensity parameters. Is needed to say that ICG is a compound that has not a stable concentration all along the surgery. It is administrated as a bolus and has its own time curves of elimination. As we apply it only in one particular moment, always 2 min after its infusion, we cannot talk about the *inflow* parameters (T_1/2_, time to peak and slope)^24^.

We consider that SERGREEN is a reliable software to traduce green images to an absolute number, which is directly related to the perfusion status of the bowel. However, it is one of its inherent limitations so it only works with those image devices that uses the green to traduce the amount of infrared light that is captured by the camera. The software can be improved to work with other color scales in the future, as grey scales.

ICG *inflow* parameters give information about the behavior of the compound after the infusion. Some features of the patient can modify ICG curves, such as portal hypertension, arterial hypertension or dose of administration, which can modify not only the maximal fluorescence readable, but also reduce the time to reach this maximum and also prolongate the time that the compound diffuses because it is more difficult to drain it from the tissues. In our particular study, these three factors were controlled so they do not produce biases on the data, which gives internal validity to our results in a pig model.

## Conclusions

The SERGREEN program allows quantification of ICG fluorescence intensity and the detection of significant differences between normal and experimental ischemia. Once 15 min has passed since the induction of ischemia, the ICG intensity can be measured at any time. A reduction in ICG fluorescence intensity of 32.6% or greater correlates with complete tissue ischemia.

## References

[1] Li Z, Zhou Y, Tian G (2021). Meta-analysis on the efficacy of indocyanine green fluorescence angiography for reduction of anastomotic leakage after rectal cancer surgery. Am. Surg..

[2] Urbanavičius L, Pattyn P, Van de Putte D, Venskutonis D (2011). How to assess intestinal viability during surgery: A review of techniques. World J. Gastrointest. Surg..

[3] Mäkelä JT, Kiviniemi H, Laitinen S (2003). Risk factors for anastomotic leakage after left-sided colorectal resection with rectal anastomosis. Dis. Colon Rectum..

[4] Sorensen LT, Jorgensen T, Kirkeby LT, Skovdal J, Vennits B, Wille-Jorgensen P (1999). Smoking and alcohol abuse are major risk factors for anastomotic leakage in colorectal surgery. Br. J. Surg..

[5] Golub R, Golub RW, Cantu R, Stein HD (1997). A multivariate analysis of factors contributing to leakage of intestinal anastomoses. J. Am. Coll. Surg..

[6] Kingham TP, Pachter HL (2009). Colonic anastomotic leak: Risk factors, diagnosis, and treatment. J. Am. Coll. Surg..

[7] Boni L, David G, Dionigi G, Rausei S, Cassinotti E (2016). Fingerhut A Indocyanine green-enhanced fluorescence to assess bowel perfusion during laparoscopic colorectal resection. Surg. Endosc..

[8] Urbanavičius L, Pattyn P, Van de Putte D, Venskutonis D (2011). How to assess intestinal viability during surgery: A review of techniques. Surg. Endosc..

[9] McCulloch P, Altman DG, Campbell WB, Flum DR, Glasziou P, Marshall JC, Nicholl J, Aronson JK, Barkun JS, Blazeby JM, Boutron IC, Campbell WB, Clavien PA, Cook JA, Ergina PL, Feldman LS, Flum DR, Maddern GJ, Nicholl J, Reeves BC, Seiler CM, Strasberg SM, Meakins JL, Ashby D, Black N, Bunker J, Burton M, Campbell M, Chalkidou K, Chalmers I, de Leval M, Deeks J, Ergina PL, Grant A, Gray M, Greenhalgh R, Jenicek M, Kehoe S, Lilford R, Littlejohns P, Loke Y, Madhock R, McPherson K, Meakins J, Rothwell P, Summerskill B, Taggart D, Tekkis P, Thompson M, Treasure T, Trohler U, Vandenbroucke J (2009). No surgical innovation without evaluation: The IDEAL recommendations. Lancet.

[10] Hirst A, Philippou Y, Blazeby J, Campbell B, Campbell M, Feinberg J, Rovers M, Blencowe N, Pennell C, Quinn T, Rogers W, Cook J, Kolias AG, Agha R, Dahm P, Sedrakyan A (2019). No surgical innovation without evaluation: Evolution and further development of the IDEAL framework and recommendations. Ann. Surg..

[11] Sherwinter DA, Gallagher J, Donkar T (2012). Intra-operative transanal near infrared imaging of colorectal anastomotic perfusion: A feasibility study. Colorectal Dis..

[12] Diana M, Noll E, Diemunsch P, Barry B, Namer IJ, Demartines N (2014). Enhanced-reality video fluorescence: A real-time assessment of intestinal viability. Ann. Surg..

[13] Moyer HR, Losken A (2012). Predicting mastectomy skin flap necrosis with indocyanine green angiography: The gray area defined. Plast. Reconstr. Surg..

[14] Galema HA, Meijer RPJ, Lauwerends LJ, Verhoef C, Burggraaf J, Vahrmeijer AL, Hutteman M, Keereweer S, Hilling DE (2021). Fluorescence-guided surgery in colorectal cancer: A review on clinical results and future perspectives. Eur. J. Surg. Oncol..

[15] Saha S, Jaiswal VK, Sharma P, Aswal DK (2020). Evolution of SI base unit candela: Quantifying the light perception of human eye. Mapan.

[16] Haslik W, Pluschnig U, Steger GG, Zielinski CC, Schrögendorfer KF, Nedomansky J, Bartsch R, Mader RM (2014). Indocyanine green video angiography predicts outcome of extravasation injuries. PLoS ONE.

[17] Otero-Piñeiro AM, de Lacy FB, Van Laarhoven JJ, Martín-Perez B, Valverde S, Bravo R, Lacy AM (2021). The impact of fluorescence angiography on anastomotic leak rate following transanal total mesorectal excision for rectal cancer: A comparative study. Surg. Endosc..

[18] Impellizzeri HG, Pulvirenti A, Inama M, Bacchion M, Marrano E, Creciun M, Casaril A, Moretto G (2020). Near-infrared fluorescence angiography for colorectal surgery is associated with a reduction of anastomotic leak rate. Updates Surg..

[19] Lütken CD, Achiam MP, Osterkamp J (2021). Quantification of fluorescence angiography: Toward a reliable intraoperative assessment of tissue perfusion: A narrative review. Langenbecks Arch. Surg..

[20] Ashraf SQ, Burns EM, Jani A, Altman S, Young JD, Cunningham C, Faiz O, Mortensen NJ (2013). The economic impact of anastomotic leakage after anterior resections in English NHS hospitals: Are we adequately remunerating them?. Colorectal Dis..

[21] Roy S, Ghosh S, Yoo A (2015). An assessment of the clinical and economic impact of establishing ileocolic anastomoses in right-colon resection surgeries using mechanical staplers compared to hand-sewn technique. Surg. Res. Pract..

[22] Villegas-Tovar E, Jimenez-Lillo J, Jimenez-Valerio V, Diaz-Giron-Gidi A, Faes-Petersen R, Otero-Piñeiro A, De Lacy FB, Martinez-Portilla RJ, Lacy AM (2020). Performance of Indocyanine green for sentinel lymph node mapping and lymph node metastasis in colorectal cancer: A diagnostic test accuracy meta-analysis. Surg. Endosc..

[23] Shirakawa S, Toyama H, Kido M, Fukumoto T (2019). A prospective single-center protocol for using near-infrared fluorescence imaging with indocyanine green during staging laparoscopy to detect small metastasis from pancreatic cancer. BMC Surg..

[24] Wada T, Kawada K, Takahashi R, Yoshitomi M, Hida K, Hasegawa S, Sakai Y (2017). ICG fluorescence imaging for quantitative evaluation of colonic perfusion in laparoscopic colorectal surgery. Surg. Endosc..

